# Low anterolateral incision for single-port extraperitoneal robot-assisted pyeloplasty: description of technique and initial experience

**DOI:** 10.1007/s00345-024-04915-4

**Published:** 2024-04-26

**Authors:** Roxana Ramos, Jaya S. Chavali, Ethan Ferguson, Nicolas Soputro, Albert Geskin, Audrey Rhee, Jihad Kaouk

**Affiliations:** 1 Glickman Urologic & Kidney Institute, Cleveland Clinic, Cleveland, OH 44195 USA; 2 WakeMed Raleigh Campus, Raleigh, NC 27610 USA

**Keywords:** Pyeloplasty, Single port, Robotic, Extraperitoneal

## Abstract

**Purpose:**

This study aims to describe the surgical steps for the single-port low anterolateral extraperitoneal approach to pyeloplasty, report its feasibility, and share the initial outcomes of our experience.

**Methods:**

We analyzed all consecutive patients who underwent single-port low anterolateral extraperitoneal pyeloplasty due to ureteropelvic junction obstruction (UPJO). The surgical steps included a pure single-port approach through a 3.5 cm low anterolateral incision two fingerbreadths above the superior pubic ramus. The ureter was localized and followed cranially, a dismembered pyeloplasty was performed, and a running ureteropelvic anastomosis was completed. No drains were placed. The urinary catheter was removed upon discharge, and the ureteral stent after 3–5 weeks.

**Results:**

A total of eight cases (two adults and six children) were completed successfully, without complications or conversions. Median operative time, console time, and estimated blood loss were 208.5 min, 114.5 min, and 10.0 ml, respectively. All patients were discharged within 24 h, except for one that required urinary output observation due to retention. There were no major postoperative complications. The median pain score at discharge was 0/10. Only one patient was prescribed PRN opioids at discharge. The readmission rate was 0.0%. All patients were asymptomatic on their last follow-up with no definitive obstruction on imaging, and no requirement for additional procedures or stents.

**Conclusion:**

Single-port low anterolateral extraperitoneal pyeloplasty is a feasible alternative for surgical treatment of UPJO in adult and pediatric patients with improved recovery outcomes.

## Introduction

Ureteropelvic junction obstruction (UPJO) can lead to impaired urinary flow, increased risk of infection, and compromised renal function if left untreated. Pyeloplasty has proven to be an effective intervention for restoring urinary flow in UPJO [1].

Due to the anatomical location of the UPJ, surgical access can be obtained through different angles, with diverse tools, and the reconstruction can be done with a variety of techniques. The open-dismembered classic technique was described in 1951 by Anderson-Hynes [2]. Although the basics of this technique are still used and preferred, in recent years, we have witnessed remarkable progress in terms of minimally invasive surgical (MIS) approaches, offering patients the benefits of reduced postoperative pain, shorter hospital stay, and faster recovery than open surgery [3, 4].

Previously described minimally invasive pyeloplasty approaches include laparoscopic (transperitoneal, transmesenteric, and retroperitoneal), multi-port (MP) robot-assisted (transperitoneal and retroperitoneal), laparoendoscopic single-site surgery (LESS) (transperitoneal), and single-port (SP) robot-assisted (transperitoneal) [5–10]. Although their results may be comparable to the standard open approach and their postoperative outcomes improved, the quest towards further minimizing MIS continues with the novel SP low anterolateral extraperitoneal approach.

The objective of this study is to describe the technique of the SP low anterolateral extraperitoneal approach, to report its feasibility, and to share our initial outcomes in adult and pediatric patients. To our knowledge, this is the first pure SP extraperitoneal pyeloplasty series published.

## Materials and methods

### Study design

With the approval of the Institutional Review Board, we collected data prospectively for all the SP pyeloplasty cases performed consecutively by one surgeon from October 2018 to February 2023. From the entire cohort (*N* = 25), we analyzed the cases that were done through the novel low anterolateral extraperitoneal approach (*N* = 8). All cases were performed by an experienced robotic surgeon with the aid of a pediatric urologist for the pertinent population.

### Patient selection

The surgical indication was UPJO cases that required intervention due to severe symptoms or hydronephrosis with impairment of kidney function. The diagnosis was confirmed by imaging preoperatively in all cases. The only exclusion criterion was an age of less than 6 months. The approach was offered as an alternative to the patient or parents establishing the potential advantages of the low anterolateral incision extraperitoneal technique, and the ultimate decision was theirs.

### Surgical technique

The novel low anterolateral extraperitoneal approach to SP pyeloplasty is a modified technique based on the previously published transperitoneal SP pyeloplasty [11]. In the following paragraphs, we describe the surgical steps according to our experience.

Following induction of general anesthesia, a cystoscopy was performed, and a ureteral stent was placed for patients who required it. A modified flank position was achieved by tilting the lower half of the body 45° to 60° towards the patient’s back.

**Fig. 1:**
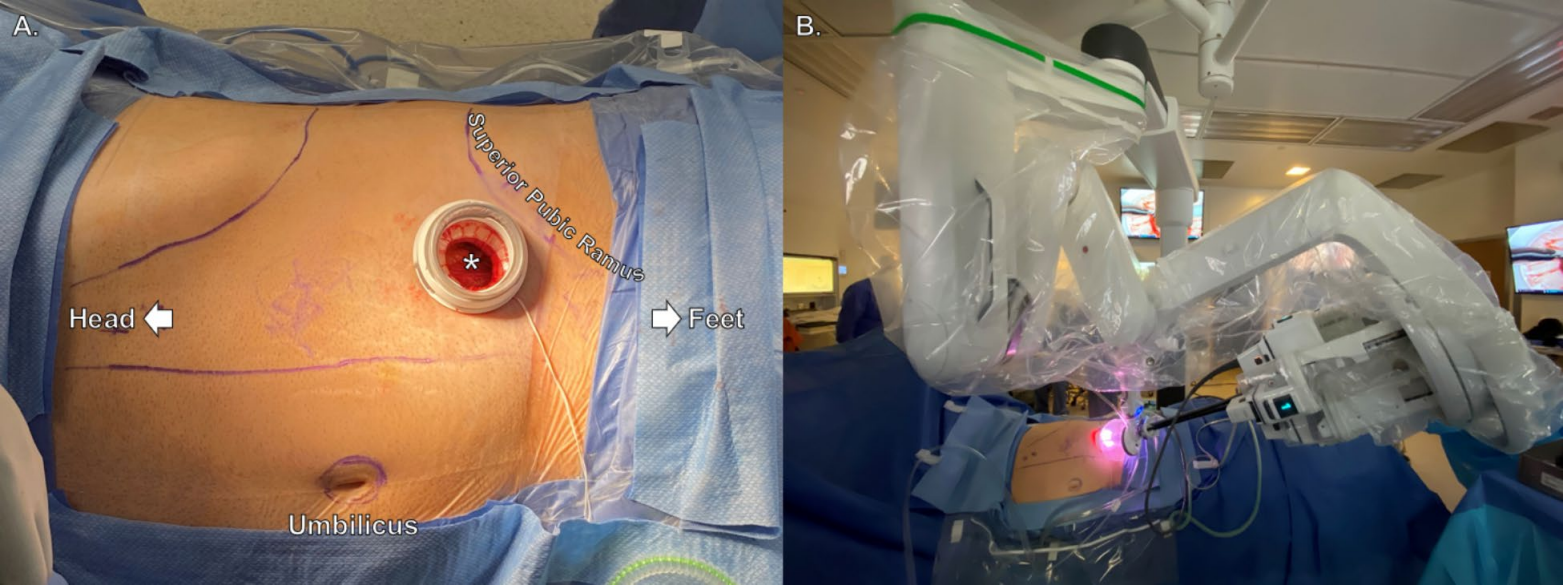
Patient positioning, low anterolateral access to the retroperitoneal space, and single-port docked robot. *Single-port low anterolateral access to the extraperitoneal space

A low anterolateral transverse incision was made two fingerbreadths above the superior pubic ramus. The incision size ranged from 2.5 to 3.5 cm according to the age of the patient. Once the skin, subcutaneous tissue, fascia, and external oblique muscle had been transected, blunt dissection was performed to develop the retroperitoneal space for the inner ring of a wound protector-retractor. Then, a small-incision da Vinci SP Access Port kit (Intuitive Surgical Inc., Sunnyvale, CA) was placed and the da Vinci SP (Intuitive Surgical Inc., Sunnyvale, CA) robot was docked (Fig. 1). The instruments we used were monopolar scissors, Cadiere forceps, and Maryland bipolar forceps. Insufflation of the retroperitoneal space was set at 10 mmHg for adults and 8 mmHg for pediatric patients using an AirSeal device (CONMED, Utica NY).

**Fig. 2:**
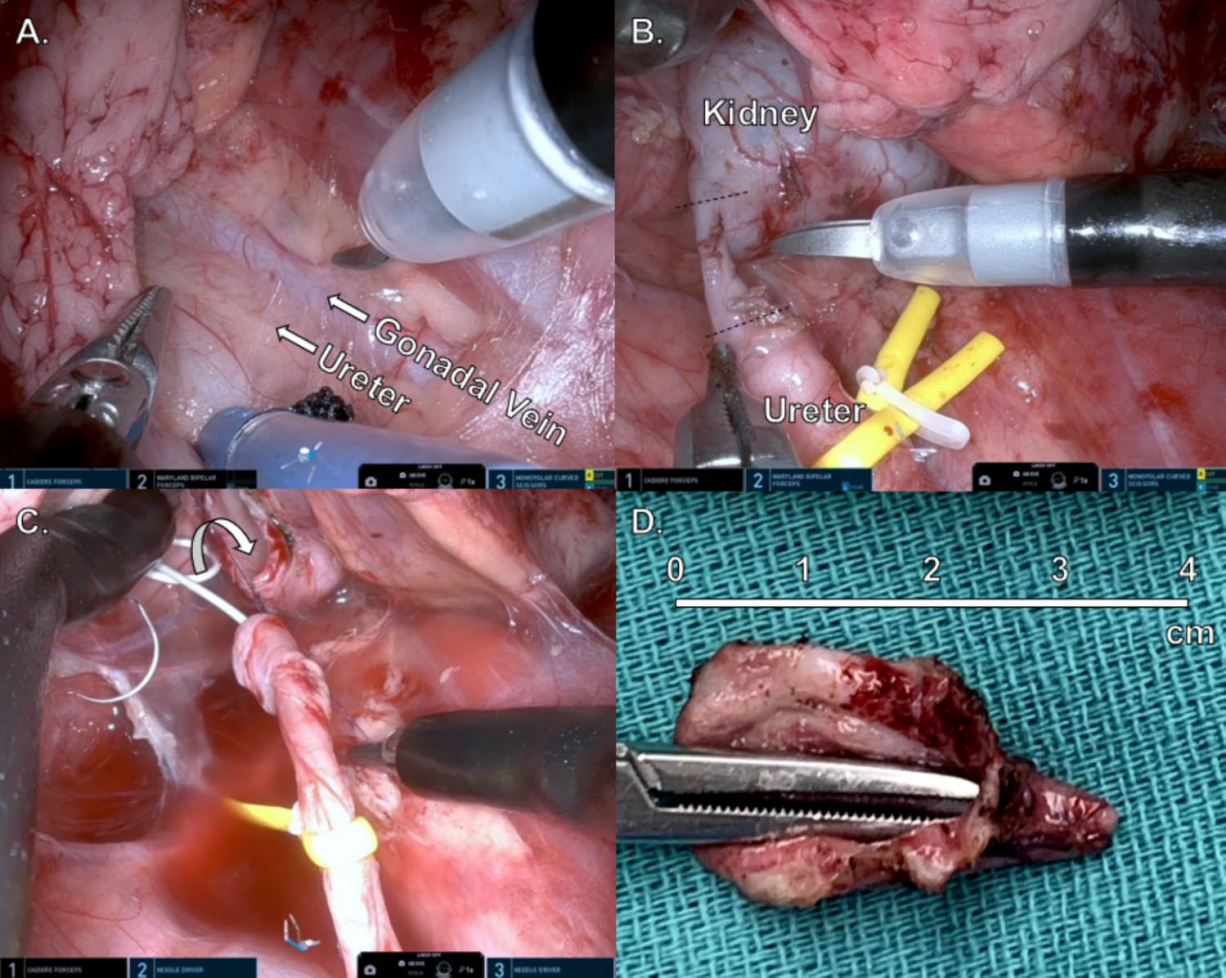
Single-port extraperitoneal dismembered pyeloplasty. *A* Retroperitoneum view. *B* Ureter transection to remove the affected segment. *C* Beginning of ureteropelvic anastomosis after dismembered pyeloplasty with pelvis reduction. *D* UPJO specimen

Dissection started by removing adhesions, visualizing the peritoneum, and following the psoas muscle cranially. Once the ureter was encountered, minimal cautery was used near it, and it was isolated with a vessel loop (Fig. 2A). Dissection continued superiorly until reaching the UPJ.

A classical dismembered pyeloplasty was done by sharply excising the stenotic segment. Pelvic reduction was performed if deemed necessary (Fig. 2B). Next, spatulation of the ureter was done laterally, and spatulation of the remainder renal pelvis medially. The specimen was retrieved through the access port. Finally, we performed a running UPJ anastomosis along the anterior and separate posterior walls using 4–0 or 5–0 Vicryl (Ethicon Inc., Raritan, NJ) sutures (Fig. 2C). Care was taken to place the pigtail end of the ureteral stent inside the renal pelvis. Once the anastomosis was completed, instruments were pulled back and the robot was undocked (Fig. 2D). The incision was closed in layers and local anesthesia was infused in the wound area. No drains were placed, and a urinary catheter was left for 0–1 days.

### Outcome measures and statistical analysis

Demographic, perioperative, and follow-up variables were collected. The FLACC scale was used to assess pain levels in nonverbal pediatric patients [12]. Surgical success was defined as asymptomatic stent-free patients at the last follow-up appointment, no obstruction on imaging, and/or good renal function on the affected side. Statistical analysis was performed using Microsoft Excel (Microsoft, Redmond, WA).

## Results

A total of eight cases were analyzed, which included two adults and six children (Table 1). Male to female ratio was 7:1. The age ranged from 1 to 59 years. The six pediatric patients had no relevant past medical or surgical histories, except for an appendectomy performed on a teenager two months before the pyeloplasty. A 32-year-old female patient had a history of recurrent urinary tract infections and four vaginal deliveries. The oldest patient (M59) had a complex metabolic and cardiovascular past medical history.

**Table 1:** Demographic, baseline characteristics, and perioperative outcomes (*N* = 8)

	Patient 1	Patient 2	Patient 3	Patient 4	Patient 5	Patient 6	Patient 7	Patient 8
Sex	Female	Male	Male	Male	Male	Male	Male	Male
Age (years)	32	16	3	1	15	59	1	5
PMH/PSH	Recurrent UTI	None	None	None	Appendectomy	CAD, COPD, DM, HLD, HTN, MDD, NASH, OSA	None	None
Symptoms	Flank Pain	Flank pain + N/V	None	None	None	None	None	Flank pain
Laterality	Right	Right	Left	Left	Left	Left	Left	Left
Renal scan functionality								
Affected kidney (%)	30	34	50	48	50	42	52	30
Unaffected kidney (%)	70	66	50	52	50	58	48	70
Etiology of UPJO	Idiopathic	Crossing Vessel	Congenital	Congenital	Idiopathic	Congenital	Congenital	Idiopathic
Preoperative stent	Yes	No	No	No	No	Yes	Yes	No
Incision size (cm)	3.5	3.5	3.5	3.0	3.5	3.5	2.5	3.5
Operative time (min)	175	203	214	220	186	173	220	235
Console time (min)	N/A	109	115	114	135	130	N/A	100
EBL (ml)	20	30	5	10	10	5	10	10
Pelvis reduction	No	No	No	Yes	Yes	Yes	Yes	Yes
Intraoperative complication	No	No	No	No	No	No	No	No
Length of stay (hours)	2.5	22.7	44.25	22.3	18.0	4.1	18.0	7.0
Pain score at discharge	0	0	0	0	2	4	0	3
Pathology report	Chronic inflammation	Chronic inflammation + fibromuscular hyperplasia	Chronic inflammation	Chronic inflammation + mucosal denudation	Chronic inflammation + fibrosis	Mucosal denudation + hemorrhage + fibrosis	Chronic inflammation	Chronic inflammation
Urinary catheter duration (days)	0	1	2	1	0	7	1	0
Ureteral stent duration (days)	26	38	28	38	38	9*	32	31

*CAD* Coronary Artery Disease, *COPD* Chronic Obstructive Pulmonary Disease, *DM* Diabetes Mellitus, *EBL* Estimated Blood Loss, *HLD* Hyperlipidemia, *HTN* Hypertension, *MDD* Major Depressive Disorder; *N/A* Not Available, *NASH* Non-Alcoholic Steatohepatitis, *N/V* Nausea and Vomiting, *OSA* Obstructive Sleep Apnea, *PMH* Past Medical History, *PMS* Past Surgical History, *UPJO* Ureteropelvic Junction Obstruction, *UTI* Urinary Tract Infection

^*^Stent was removed early in another center, replacement or a new stent later on was not required

Out of the eight, five patients were asymptomatic (62.5%) on diagnosis, and the other three patients presented with ipsilateral flank pain ± nausea/vomiting. Patients were diagnosed with UPJO via imaging studies (ultrasound, retrograde pyelogram, and/or CT scan). In addition, all patients had a preoperative renal scan that showed > 30% of renal function of the affected kidney. The etiology of UPJO was distributed as follows: congenital (50%), idiopathic (37.5%), and crossing vessel (12.5%). One of the infants had a duplicated left renal system with high-grade lower moiety UPJO. Most of the cases were left-sided (75%), and three patients (37.5%) had ureteral stents prior to the surgery.

All cases were first-time pyeloplasty and completed successfully without the need for additional ports or conversion. The incision size for SP access was 3.5 cm for all except the infants (2.5 cm and 3.0 cm). Median operative time, console time, and estimated blood loss were 208.5 min (IQR 183.2–220.0), 114.5 min (IQR 110.2–126.2), and 10 ml (IQR 8.7–12.5), respectively. There were no incidental crossing vessels seen during the procedures. There were no intraoperative complications.

All the patients were discharged within 24 h, except for one that required observation of urine output following catheter reinsertion for urinary retention. There were no other deviations from the expected postoperative course and no major postoperative complications. Urinary catheters were removed before discharge in seven of the eight cases, and the oldest patient had the catheter for seven days. The median pain score at discharge was 0/10. Only one patient was prescribed PRN opioids at discharge. The readmission rate was 0%. All pathological specimens were benign, showing chronic inflammation, fibromuscular hyperplasia, mucosa denudation, and/or fibrosis.

Ureteral stents were removed between 3 and 5 weeks after the surgery. All patients were asymptomatic on their last follow-up with no definitive obstruction on imaging, and no requirement for additional procedures or stents. The median follow-up time was 2 months.

## Discussion

In this study, we present the description of the novel low anterolateral extraperitoneal approach for SP dismembered pyeloplasty and report the outcomes of the first eight consecutive cases done in our center. In recent years, many different surgical approaches have been published to treat UPJO, seeking minimal manipulation, standard outcomes, and fast recovery, especially because the indication for treatment is frequently seen in the pediatric population. From open to laparoscopic and robotic, pyeloplasty success rates have all reached the high 90.0% to 100.0% [1, 13, 14]; however, the advantages of MIS include smaller incisions and delicate manipulation of tissues that grant less operative pain and a faster recovery, which promotes a more rapid return to normal activities [3, 15]. Furthermore, it has been demonstrated that MP robot-assisted approaches are superior to laparoscopic in terms of a shorter learning curve, decreased operative times, shorter duration of hospital stay, and lower complication rates [4]. Despite these numbers, conventional laparoscopic pyeloplasty has persisted because of high-cost robotic systems and some satisfactory reported results [16].

Another important aspect to consider when choosing a technique for MIS is the approach. Pyeloplasty can be performed via transperitoneal, transmesenteric, or retroperitoneal (extraperitoneal) approaches. Although the transperitoneal approach has the advantage of familiar anatomy, regionalizing the surgery to the location of the disease has added benefits. Transperitoneal and retroperitoneal approaches have been compared to each other in laparoscopic pyeloplasty in children, demonstrating statically significant shorter operative times, median hospital stay, and time to oral feeding after surgery with the retroperitoneal approach [17]. We did not find a study comparing transperitoneal vs retroperitoneal with robotic systems. However, we hypothesize that the advantages are mirrored since avoidance of the intraperitoneal space is known to enhance recovery in other types of urological surgeries [18]. As for an anterior-incision extraperitoneal approach, to our knowledge, there is only one case report published for a laparoscopic pyeloplasty for a horseshoe kidney case and data on two patients in a series of SP retroperitoneal cases [19, 20].

The SP platform is a low-profile robot that allows total rotation and relocation of the boom, and a 24 cm reach of instruments, ideal for retroperitoneal and multi-quadrant surgeries. One of the factors that have recently enhanced SP surgery is the use of the floating-dock technique with the purpose-built da Vinci SP Access Port, which allows a bigger working area and less insufflation pressure [21].

The use of the SP robot for transperitoneal pyeloplasty was first described by Agarwal et al. [22] and Lenfant et al. [11] in 2020. Since then, other SP transperitoneal series have been published with a variety of modifications, including transumbilical or low anterior midline incisions, and additional ports [23–25]. However, the technique has evolved rapidly in the last 3 years seeking to improve cosmetic outcomes and avoid violating the peritoneum. Our technique is purely SP, with no additional ports. Furthermore, the anterolateral location of the incision facilitates direct extraperitoneal access and decreases the need for medial bowel retraction. In our experience, there is no need for postoperative drains, just a urinary catheter for a day and a ureteral stent for four weeks. The surgical success rate in our series was 100.0%, with promising immediate postoperative outcomes, and potential for pyeloplasty as a standard outpatient procedure. A recently published series by Pellegrino et al. [20] highlights the early postoperative advantages of the SP retroperitoneal approach, including low complications rate, mild postoperative pain, and feasibility of same-day discharge.

**Fig. 3:**
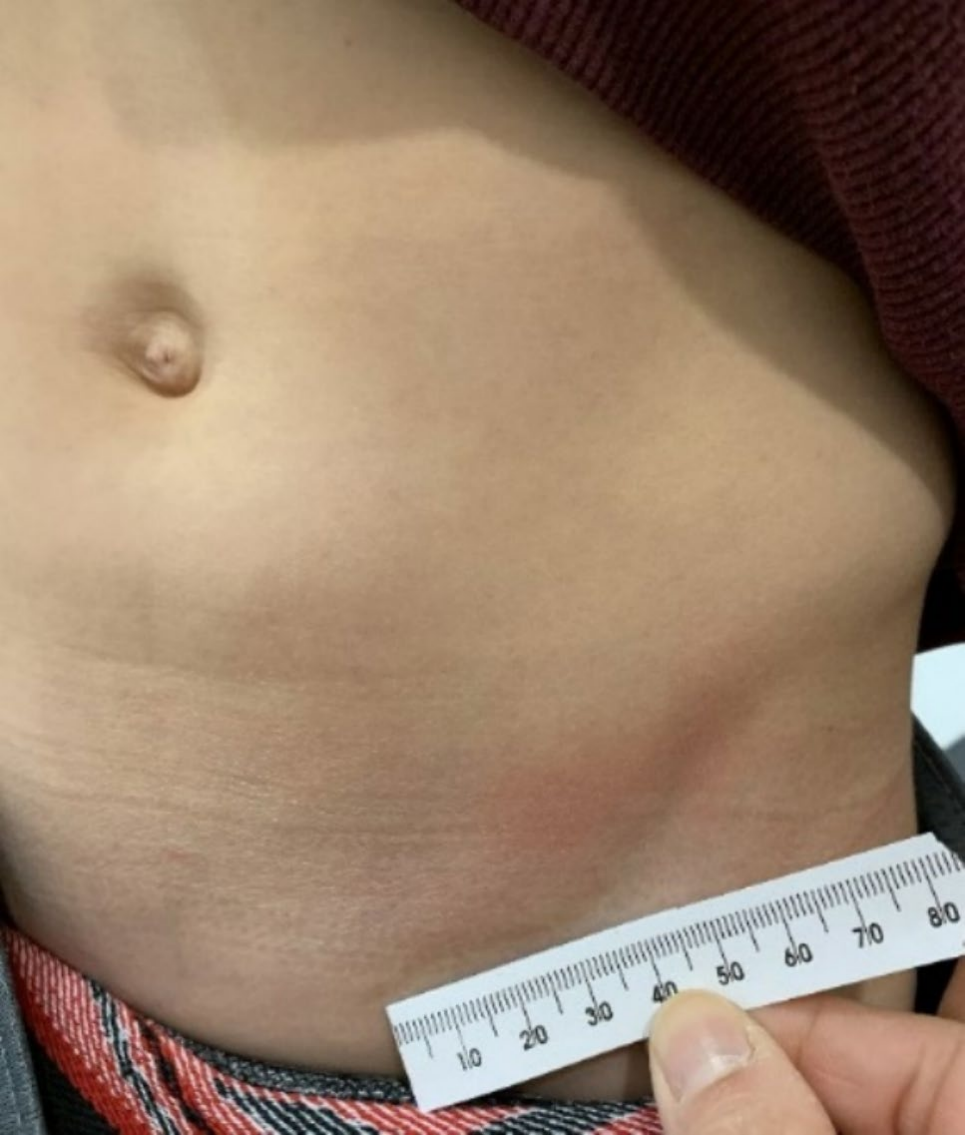
Follow-up incision site in pediatric patient

When comparing robot-assisted pyeloplasty techniques, a series published in 2022 by Beksac et al. [26] reported a shorter length of stay, and lower opioid prescription usage with the SP, pain scores were comparable between MP and SP. Furthermore, a meta-analysis published in June 2023 by Gu et al. [27] compared SP vs MP pyeloplasty and found that SP was associated with shorter hospital stay duration, less postoperative pain, and better cosmetic appearance. Neither of these studies found statistical differences regarding operative time, EBL, complications, or recovery of renal function. Finally, we would like to highlight that the transition from MP to SP allows the benefit of a single hidden scar (Fig. 3), as similarly described by Gargollo [28]. While cosmetic results are not usually the priority, a large scar or multiple visible scars may affect a child’s mental health when exposed to peers.

In our center, SP transperitoneal pyeloplasty was first done in 2018, and it was the approach used until 2021. After this point, all cases were attempted via the retroperitoneal route, excluding patients for whom anatomical characteristics did not allow a retroperitoneal approach. When comparing SP transperitoneal vs extraperitoneal, the latter shows a relatively longer length of stay (12 vs 18 h), less level of pain at discharge (2.8/10 vs 0/10), and similar opioid-free prescription (~ 90.0%) [26]. However, we suggest that a matching of the population should be performed to make the results comparable.

Several limitations must be acknowledged in interpreting the findings of this study. First, the study was conducted at a single center, which may restrict the generalizability of the results to other healthcare settings. Second, while the procedures were performed by an experienced robotic surgeon, it is important to note that individual surgeon expertise and skill level can impact surgical outcomes, especially with the novel SP robotic platform. Furthermore, the study population consisted of a small, heterogeneous series of patients with a median follow-up of 2 months, which may not capture long-term complications or assess the durability of the surgical intervention.

Future studies should be done to compare larger series of the SP low anterolateral extraperitoneal pyeloplasty to other UPJO surgical approaches.

## Conclusion

SP low anterolateral extraperitoneal pyeloplasty is a feasible alternative for surgical treatment of UPJO in adult and pediatric patients that further advances the field of MIS. The SP extraperitoneal approach echoes the postoperative benefits of other robotic techniques, such as fast recovery and minimal postoperative pain.

## Data Availability

Data is not publically available, but it may be shared upon request.
